# Reduced‐dose versus standard‐dose total body irradiation before allogeneic haematopoietic stem cell transplantation in adults with acute lymphoblastic leukaemia

**DOI:** 10.1111/bjh.70835

**Published:** 2026-09-15

**Authors:** Isabella Gruber, Matthias Edinger, Hendrik Poeck, Elisabeth Meedt, Ernst Holler, Wolfgang Herr, Oliver Koelbl, Daniel Wolff

**Affiliations:** ^1^ Department of Radiation Oncology University Hospital Regensburg Regensburg Germany; ^2^ Department of Internal Medicine III University Hospital Regensburg Regensburg Germany; ^3^ Leibniz Institute for Immunotherapy Regensburg Germany

**Keywords:** acute lymphoblastic leukaemia, allogeneic haematopoietic stem cell transplantation, conditioning, non‐relapse mortality, relapse, total body irradiation

## Abstract

Comparative data on reduced‐dose (8 Gy) versus standard‐dose (12 Gy) total body irradiation (TBI) before allogeneic haematopoietic stem cell transplantation (allo‐HSCT) for adults with acute lymphoblastic leukaemia (ALL) remain limited. We retrospectively analysed 101 adults undergoing first allo‐HSCT between 1999 and 2024 after 8 Gy or 12 Gy fractionated TBI‐based conditioning. Relapse and non‐relapse mortality (NRM) were analysed using multivariable Fine–Gray models. Median follow‐up was 8.1 years. Although the 8 Gy cohort was older, had greater comorbidity burden and a higher‐risk disease profile, unadjusted NRM, relapse incidence and overall survival did not differ between groups. In multivariable analyses, 12 Gy TBI was associated with increased NRM (sub‐distribution hazard ratio 5.97, 95% confidence interval [CI] 1.77–20.15; *p* = 0.004) without evidence of reduced relapse incidence. The association between 12 Gy TBI and higher NRM was consistent across sensitivity analyses, including adjustment for transplant year and conditioning regimen, cause‐specific Cox regression and propensity score overlap weighting. The association of 12 Gy TBI with inferior overall survival in the multivariable model was not confirmed after overlap weighting. Consistent pre‐transplant measurable residual disease data were unavailable, limiting adjustment for this prognostic factor. These findings support prospective evaluation of reduced‐dose TBI in selected adults with ALL.

## INTRODUCTION

Allogeneic haematopoietic stem cell transplantation (allo‐HSCT) remains a potentially curative treatment for adults with high‐risk acute lymphoblastic leukaemia (ALL). Total body irradiation (TBI)‐based conditioning remains a cornerstone in this setting because of its broad anti‐leukaemic activity and ability to achieve cytoreduction in sanctuary sites such as the central nervous system and testes.^1^, ^2^ Consequently, TBI‐containing regimens remain widely used despite advances in chemotherapy, targeted therapies and measurable residual disease‐guided treatment strategies.

Although fractionated 12 Gy TBI has long been considered the reference myeloablative approach for younger and fit adults with ALL, the optimal radiation dose remains uncertain.^2^, ^3^, ^4^ Reduced‐dose regimens, particularly 8 Gy TBI, are mainly used in older or comorbid patients to reduce toxicity, resulting in selection differences between dose groups. Whether standard‐dose (12 Gy) TBI provides sufficient improvement in leukaemia control to justify a potential increase in non‐relapse mortality (NRM) remains unresolved. Evidence directly comparing reduced‐ and standard‐dose TBI in adult ALL is limited to retrospective studies, with prospective comparative data lacking.^4^

This question has gained importance with advances in transplantation practice. Improvements in donor selection, supportive care and graft‐versus‐host disease (GVHD) prophylaxis have reduced transplantation‐related mortality, prompting re‐evaluation of conditioning intensity versus toxicity. The largest contemporary registry analysis found no improvement in relapse control or survival with 12 Gy TBI, but patient‐level data on toxicity, conditioning backbone and long‐term complications were limited.^4^

We retrospectively analysed adults undergoing first allo‐HSCT for ALL with standardized fractionated 8 or 12 Gy TBI‐based conditioning. The primary objective was to compare relapse incidence and NRM using competing‐risk methodology, with multivariable models and sensitivity analyses applied to account for relevant differences in baseline characteristics and temporal changes in transplant practice. Secondary objectives included overall survival (OS), progression‐free survival (PFS), GVHD and secondary malignancies.

## MATERIALS AND METHODS

### Data collection

We performed a retrospective single‐centre cohort study of consecutive adults with ALL undergoing first TBI‐based allo‐HSCT at the University Hospital Regensburg between January 1999 and March 2024. Eligible patients received fractionated 8 Gy or 12 Gy TBI before first allo‐HSCT from matched sibling, matched or mismatched unrelated, haploidentical or mismatched related donors. Patients receiving non‐TBI‐based conditioning (*n* = 6) or 4 Gy TBI (*n* = 2) were excluded. A total of 101 patients met the inclusion criteria.

Selection of conditioning intensity was based on institutional practice and physician discretion, considering patient age, comorbidity burden and disease characteristics. Patient aged >55–60 years and a Hematopoietic Cell Transplantation‐Comorbidity Index (HCT‐CI) >3 generally received 8 Gy TBI. Clinical data were extracted from institutional electronic medical records. Extracted variables included patient, disease, donor, transplant and outcome characteristics. Follow‐up was complete through July 2025.

The study was approved by the Ethics Committee of the University of Regensburg (reference number 20‐1810‐101; approval date: 22 April 2020). All patients provided written informed consent.

### Definitions and statistical end‐points

The primary end‐point was the cumulative incidence of NRM, with relapse of ALL considered a competing risk. Secondary end‐points included the cumulative incidence of relapse (CIR), grade II–IV acute GVHD, chronic GVHD requiring systemic immunosuppression, PFS and OS. Additional analyses included causes of death and secondary solid malignancies.

All time‐to‐event outcomes were calculated from the date of allo‐HSCT. NRM was defined as death from any cause in the absence of prior relapse, with relapse considered a competing event. Relapse was defined as haematological recurrence requiring treatment. Isolated mixed chimerism or molecular detection of minimal residual disease not necessitating intervention was not classified as relapse. For CIR, NRM was considered a competing event.

Acute and chronic GVHD were classified according to established consensus criteria.^5^, ^6^ Grade II–IV acute and chronic GVHD requiring systemic treatment were analysed using competing‐risk methodology.

PFS was defined as the interval from allo‐HSCT to relapse, progression or death from any cause. OS was defined as the interval from allo‐HSCT to death. Patients without events were censored at last follow‐up.

Disease status at transplantation was categorized as first complete remission (CR1) or beyond first complete remission (>CR1). ALL risk groups were defined by cytogenetic and molecular features. High‐risk ALL included t(9;22)(q34;q11.2) (Philadelphia chromosome, *BCR*::*ABL1*), t(v;11)(q21;q23) (*KMT2A* rearrangement), hypodiploidy (<44 chromosomes), complex karyotype (>3 abnormalities) and Philadelphia (Ph)‐like ALL. Favourable‐risk ALL included t(12;21)/*ETV6::RUNX1* or hyperdiploid disease (≥50 chromosomes). Intermediate‐risk ALL comprised all remaining patients.

Standardized consensus definitions for haematopoietic recovery, graft failure and donor chimerism were applied.^7^ Chimerism was assessed regularly by polymerase chain reaction (PCR)‐based analysis of peripheral blood. Full donor chimerism was defined as ≥99% donor cells. Patients dying within 28 days post‐allo‐HSCT were excluded from engraftment analysis. Causes of death were categorized hierarchically as relapse, GVHD, infection or other causes according to established transplant reporting recommendations.^8^ For exploratory analyses, conditioning regimens were categorized as fludarabine‐containing (fludarabine/TBI or cyclophosphamide/fludarabine/TBI) versus cyclophosphamide‐ or etoposide‐containing (cyclophosphamide/TBI or etoposide/TBI).

### Radiotherapy

All conditioning regimens are summarized in Table S1. TBI was delivered at the Department of Radiation Oncology, University Hospital Regensburg, using Siemens Primus linear accelerators between 1999 and 2013 and Elekta Synergy™ linear accelerators thereafter. Both systems provided comparable dose distributions and treatment parameters.^9^ Conventional TBI was delivered with 6 MV photons at extended source‐to‐surface distance in twice‐daily 2 Gy fractions separated by at least 6 h. Patients were treated in supine and prone position. A 1‐cm Makrolon® polycarbonate plate was placed above the patient to compensate for the photon skin‐sparing effect and support lung shields. For total doses >8 Gy, individualized computed tomography (CT)‐based lung shielding limited the cumulative central lung dose to 7 Gy; shielded thoracic regions were supplemented with electrons. Each position required eight rotational arcs for dose delivery. The average treatment time was 50–60 min per position, with an overall dose rate of 4 cGy/min. Additional fixed cranial and caudal beams compensated for dose variations due to the inverse square law. In vivo dosimetry at multiple body sites confirmed dose uniformity.^9^

Fifty‐six patients (55.4%) additionally received whole‐brain radiotherapy during induction according to the German Multicenter Study Group for Adult Acute Lymphoblastic Leukemia (GMALL) protocol.

### Statistical analysis

Baseline characteristics were summarized as medians with interquartile ranges (IQR) or frequencies with percentages and compared using the Mann–Whitney *U* or chi‐squared test, as appropriate.

OS and PFS were estimated using the Kaplan–Meier method and compared using log‐rank tests. Median follow‐up was estimated using the reverse Kaplan–Meier method.^10^

Cumulative incidence functions (CIF) were estimated for CIR, NRM, acute GVHD, chronic GVHD and secondary malignancies, accounting for competing risks and compared using Gray's test.^11^ CIF estimates at prespecified time points were reported with 95% confidence intervals (CIs).

Associations with competing‐risk end‐points were evaluated using Fine–Gray proportional sub‐distribution hazards models.^12^ Covariates were selected a priori based on clinical relevance and outcome‐specific considerations. Pre‐transplant measurable residual disease (MRD) status was not included as a covariate because consistent MRD data were unavailable across the study period. GVHD prophylaxis was included in the models of acute and chronic GVHD but omitted from OS, PFS, relapse and NRM models to limit model complexity given the number of events.

Exploratory interaction analyses were performed by adding interaction terms between TBI dose and each covariate included in the respective multivariable model. Global likelihood‐ratio tests assessed the overall significance of the interaction terms, and individual interaction estimates were examined separately.

Sensitivity analyses additionally adjusted for transplant year as a continuous covariate to account for temporal changes in transplantation practice. Potential confounding by conditioning regimen was explored by additionally adjusting for fludarabine‐containing versus cyclophosphamide‐ or etoposide‐containing regimens. Propensity score overlap weighting was performed to estimate the average treatment effect in the overlap population (ATO) using logistic regression including age, ALL risk group, disease status at transplantation, HCT‐CI and transplant year as covariates. Covariate balance was assessed using absolute standardized mean differences, with values <0.1 considered indicative of adequate balance. Associations between TBI dose and NRM and relapse were estimated using Fine–Gray regression and associations with OS and PFS using Cox regression, incorporating the ATO weights.

PFS and OS were analysed using Cox proportional hazards regression. Cause‐specific Cox models for relapse and NRM, using the same prespecified covariates and additional adjustment for transplant year, were performed as sensitivity analyses. The proportional hazards assumption was assessed using Schoenfeld residuals and was not violated in the final multivariable models.

Effect estimates are reported as sub‐distribution hazard ratios (SDHRs) or cause‐specific hazard ratios (CSHRs) with 95% CI. All tests were two‐sided, with *p*‐value <0.05 considered statistically significant. Analyses were performed using R version 4.5.0 (R Foundation for Statistical Computing, Vienna, Austria).

## RESULTS

### Patient characteristics and unadjusted outcomes

Table 1 summarizes baseline characteristics of the study cohort (*N =* 101). Median follow‐up was 8.1 years (IQR 5.3–12.3). Patients receiving 8 Gy TBI were older (median age, 51.4 vs. 33.6 years, *p* < 0.001), had higher comorbidity burden (HCT‐CI score 0: 17.6% vs. 52.0%; *p* < 0.001), older donors (38.0 vs. 31.0 years; *p* = 0.017) and more frequently had high‐risk ALL (60.8% vs. 32.0%; *p* = 0.005). Among patients with Philadelphia chromosome‐positive ALL (*n* = 29), pre‐transplant tyrosine kinase inhibitor (TKI) therapy was administered to 19/19 (100%) and 9/10 (90%) patients in the 8 Gy and 12 Gy groups, respectively, whereas post‐transplant TKI maintenance was similarly distributed (4/19 vs. 2/10; Table S2). Patients receiving 12 Gy underwent transplantation earlier than those receiving 8 Gy TBI (median year 2009 [IQR 2005–2017] vs. 2015 [IQR 2010–2020]; *p* = 0.034).

**TABLE 1:** Patient and transplant characteristics.

Characteristic	Category	All (*N* = 101)	TBI 8 Gy (*n* = 51)	TBI 12 Gy (*n* = 50)	*p*‐value
Follow‐up, years	Median (IQR)	8.1 (5.3–12.3)	6.5 (4.3–9.4)	9.1 (5.5–15.6)	0.058
Patient age, years	Median (IQR)	38.8 (30.6–51.4)	51.4 (42.4–57.4)	33.6 (26.4–38.0)	<0.001
Sex	Male	55 (54.5%)	24 (47.1%)	31 (62.0%)	0.163
	Female	46 (45.5%)	27 (52.9%)	19 (38.0%)	
Diagnosis	B‐cell ALL	74 (73.3%)	40 (78.4%)	34 (68.0%)	0.267
	T‐cell ALL	27 (26.7%)	11 (21.6%)	16 (32.0%)	
Philadelphia chromosome	Ph+ ALL	29 (28.7%)	19 (37.3%)	10 (20.0%)	0.078
	Ph– ALL	72 (71.3%)	32 (62.7%)	40 (80.0%)	
ALL risk groups	Fav./interm.‐risk	54 (53.5%)	20 (39.2%)	34 (68.0%)	0.005
	High‐risk	47 (46.5%)	31 (60.8%)	16 (32.0%)	
HCT‐CI	0	35 (34.7%)	9 (17.6%)	26 (52.0%)	<0.001
	1–2	39 (38.6%)	24 (47.1%)	15 (30.0%)	
	≥3	27 (26.7%)	18 (35.3%)	9 (18.0%)	
KPS	Median (IQR)	90 (80–90)	90 (80–90)	90 (80–90)	0.049
Disease status	CR1	61 (60.4%)	33 (64.7%)	28 (56.0%)	0.125
	CR2, PR1	28 (27.7%)	10 (19.6%)	18 (36.0%)	
	>CR2/Active	12 (11.9%)	8 (15.7%)	4 (8.0%)	
Disease status	CR1	61 (60.4%)	33 (64.7%)	28 (56.0%)	0.419
	>CR1	40 (39.6%)	18 (35.3%)	22 (44.0%)	
Donor types	MSD	23 (22.8%)	10 (19.6%)	13 (26.0%)	0.527
	MUD	37 (36.6%)	17 (33.3%)	20 (40.0%)	
	MMUD, MMRD	34 (33.7%)	19 (37.3%)	15 (30.0%)	
	Haploidentical	7 (6.9%)	5 (9.8%)	2 (4.0%)	
Stem cell source	Peripheral blood	86 (85.1%)	42 (82.4%)	44 (88.0%)	0.577
	Bone marrow	15 (14.9%)	9 (17.6%)	6 (12.0%)	
Donor sex	Male	74 (73.3%)	40 (78.4%)	34 (68.0%)	0.267
	Female	27 (26.7%)	11 (21.6%)	16 (32.0%)	
Donor age, years	Median (IQR)	35.0 (27.0–42.0)	38.0 (30.5–45.5)	31.0 (26.0–41.0)	0.017
Female→male D/R	Yes	11 (10.9%)	3 (5.9%)	8 (16.0%)	0.122
	No	90 (89.1%)	48 (94.1%)	42 (84.0%)	
CMV‐serostatus (D/R)	D−/R−	49 (48.5%)	26 (51.0%)	23 (46.0%)	0.731
	D−/R+	16 (15.8%)	6 (11.8%)	10 (20.0%)	
	D+/R+	20 (19.8%)	11 (21.6%)	9 (18.0%)	
	D+/R−	16 (15.8%)	8 (15.7%)	8 (16.0%)	
GVHD prophylaxis	CyA/MTX	74 (73.3%)	35 (68.6%)	39 (78.0%)	0.425
	CyA/MMF, Tac/MMF	12 (11.9%)	6 (11.8%)	6 (12.0%)	
	Post‐Tx Cy/Tac/MMF	15 (14.9%)	10 (19.6%)	5 (10.0%)	
ATG	Yes	64 (63.4%)	33 (64.7%)	31 (62.0%)	0.838
	No	37 (36.6%)	18 (35.3%)	19 (38.0%)	

Abbreviations: ATG, anti‑thymocyte globulin; CMV, cytomegalovirus; CR1, first complete remission; CyA/MMF, cyclosporine and mycophenolate mofetil; CyA/MTX, cyclosporine and methotrexate; disease status at transplantation; fav./interm.‐risk ALL, favourable‐risk ALL, t(12;21)/*ETV6*::*RUNX1* or hyperdiploidy (≥50 chromosomes); Female→male D/R, female donor to male recipient; GVHD, graft‑versus‑host disease; HCT‐CI, Hematopoietic Cell Transplantation‑Comorbidity Index; high‐risk ALL, t(9;22)(q34;q11.2) (Philadelphia chromosome, *BCR*::*ABL1*), t(v;11)(q21;q23) (*KMT2A* rearrangement), hypodiploidy (<44 chromosomes), complex karyotype (>3 chromosomal abnormalities) or Ph‑like ALL (Philadelphia chromosome‐like gene expression); intermediate‐risk ALL, patients without high‐risk or favourable abnormalities (e.g. normal karyotype, balanced translocations); KPS, Karnofsky performance status; MMRD, mismatched related donor; MMUD, mismatched unrelated donor; MSD, matched sibling donor; MUD, matched unrelated donor; post‐transplant Cy/Tac/MMF, post‐transplant cyclophosphamide, tacrolimus and mycophenolate mofetil (primarily used in haploidentical transplantation); PR1, first partial remission; Tac/MMF, tacrolimus and mycophenolate mofetil; TBI, total body irradiation.

Engraftment and donor chimerism did not differ between groups (Table S3). In unadjusted analyses, no significant differences were observed between TBI dose groups in CIR (Gray's test, *p* = 0.204), NRM (Gray's test, *p* = 0.509), acute GVHD (Gray's test, *p* = 0.731), chronic GVHD (Gray's test, *p* = 0.538), OS (log‐rank test, *p* = 0.907) or PFS (log‐rank test, *p* = 0.564). Cumulative incidence estimates at predefined time points are shown in Table 2.

**TABLE 2:** Unadjusted cumulative incidence and survival estimates at predefined time points.

	Time point	All (*N* = 101)	TBI 8 Gy (*n* = 51)	TBI 12 Gy (*n* = 50)	*p*‐value
NRM	100‐day	3% (95% CI 1–6)	—	6% (95% CI 1–13)	0.077
	2‐year	18% (95% CI 11–26)	18% (95% CI 9–29)	18% (95% CI 9–30)	0.959
	5‐year	21% (95% CI 14–30)	20% (95% CI 10–32)	23% (95% CI 12–35)	0.760
CIR	2‐year	25% (95% CI 17–34)	29% (95% CI 18–42)	20% (95% CI 10–32)	0.277
	5‐year	27% (95% CI 19–36)	32% (95% CI 19–45)	22% (95% CI 12–35)	0.309
Grade II–IV aGVHD	100‐day	48% (95% CI 37–57)	47% (95% CI 33–60)	48% (95% CI 33–61)	0.926
Chronic GVHD	2‐year	43% (95% CI 33–52)	45% (95% CI 31–58)	40% (95% CI 26–54)	0.626
	5‐year	45% (95% CI 35–55)	49% (95% CI 35–63)	40% (95% CI 26–54)	0.365
OS	2‐year	63% (95% CI 54–73)	63% (95% CI 51–77)	64% (95% CI 51–78)	0.918
	5‐year	55% (95% CI 46–66)	51% (95% CI 39–67)	59% (95% CI 46–75)	0.436
PFS	2‐year	58% (95% CI 49–69)	54% (95% CI 42–70)	63% (95% CI 51–78)	0.344
	5‐year	52% (95% CI 43–64)	49% (95% CI 37–66)	56% (95% CI 43–72)	0.503

Abbreviations: aGVHD, acute graft‐versus‐host disease; chronic GVHD, chronic GVHD requiring systemic immunosuppression; CIR, cumulative incidence of relapse; NRM, non‐relapse mortality; OS, overall survival; PFS, progression‐free survival; TBI, total body irradiation.

Figure 1 illustrates the unadjusted CIR and NRM, stratified by TBI dose. Given the baseline differences between TBI groups, adjusted multivariable analyses were prespecified as the primary analyses for assessing the association between TBI dose and outcomes.

**FIGURE 1:**
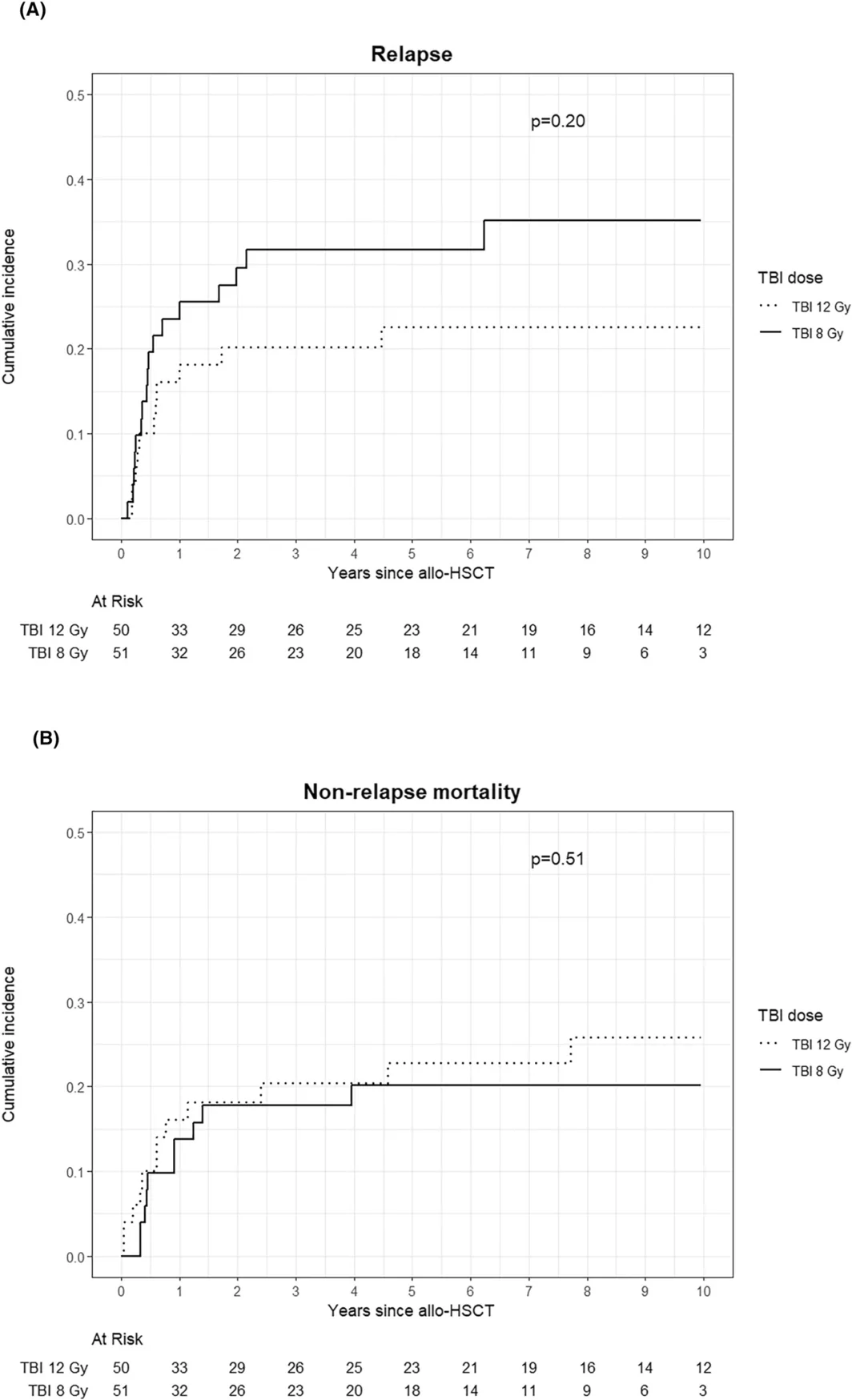
Unadjusted cumulative incidences of relapse (A) and non‐relapse mortality (B). Relapse and non‐relapse mortality were analysed as competing‐risk end‐points, and cumulative incidences were compared using Gray's test.

Isolated marrow relapse (*n* = 19, 67.9%) was the most frequent type of relapse, while five patients (17.9%) relapsed in both marrow and extra‐medullary sites. Relapse occurred in one patient each (3.6%) as central nervous system (CNS)‐only, isolated extra‐medullary, marrow plus cerebrospinal fluid or combined marrow, extra‐medullary and cerebrospinal fluid involvement.

Causes of death (*n* = 48) were equally distributed between relapse and NRM (each 50%, *n* = 24), with GVHD accounting for the majority of NRM‐related deaths (*n* = 14, 58%), followed by infections (*n* = 7, 29%) and other causes (*n* = 3, 13%). Causes of death did not differ between TBI groups (*p* = 0.387) (Table S4).

### Primary multivariable analyses

In multivariable Fine–Gray analysis of NRM and relapse (Table 3), 12 Gy TBI was independently associated with a higher sub‐distribution hazard of NRM (SDHR 5.97, 95% CI 1.77–20.15; *p =* 0.004) compared with 8 Gy TBI, without evidence of reduced relapse incidence. NRM was also associated with transplantation beyond first complete remission (CR1) (SDHR 2.83, 95% CI 1.21–6.62; *p =* 0.017) and increasing donor age (SDHR per year 1.10, 95% CI 1.05–1.16; *p* < 0.001), while relapse incidence was associated with high‐risk ALL (SDHR 2.53, 95% CI 1.11–5.77; *p =* 0.028) and transplantation beyond CR1 (SDHR 2.98, 95% CI 1.43–6.19; *p =* 0.003). Interaction analyses between TBI dose and covariates are shown in Table S5.

**TABLE 3:** Multivariable Fine–Gray regression analyses of relapse incidence and non‐relapse mortality.

Variable	Category	Relapse, SDHR (95% CI)	*p*‐value	NRM, SDHR (95% CI)	*p*‐value
TBI dose	8 Gy (ref.)	1.00		1.00	
	12 Gy	0.54 (0.21–1.37)	0.190	5.97 (1.77–20.15)	0.004
Patient age	Per 1‐year increase	0.97 (0.95–1.00)	0.058	1.02 (0.98–1.06)	0.270
Risk group	Fav./interm.‐risk (ref.)	1.00		1.00	
	High‐risk	2.53 (1.11–5.77)	0.028	1.51 (0.58–3.97)	0.400
Disease status	CR1 (ref.)	1.00		1.00	
	>CR1	2.98 (1.43–6.19)	0.003	2.83 (1.21–6.62)	0.017
HCT‐CI Score	0 (ref.)	1.00		1.00	
	1–2	1.40 (0.57–3.46)	0.470	2.23 (0.59–8.52)	0.240
	≥3	1.03 (0.30–3.54)	0.960	4.18 (1.13–15.44)	0.032
ATG	No (ref.)	1.00		1.00	
	Yes	0.83 (0.28–2.50)	0.740	1.43 (0.46–4.48)	0.540
Donor type	MSD (ref.)	1.00		1.00	
	MUD	0.42 (0.13–1.31)	0.140	1.39 (0.37–5.22)	0.630
	MMUD, MMRD	0.65 (0.17–2.47)	0.520	1.94 (0.54–6.96)	0.310
	Haploidentical	0.67 (0.15–2.95)	0.600	1.53 (0.09–25.30)	0.770
Donor age	Per 1‐year increase	1.01 (0.98–1.04)	0.490	1.10 (1.05–1.16)	<0.001

*Note*: Exploratory interaction analyses assessed whether the association between TBI dose and outcome varied according to covariates included in the respective multivariable models. Global likelihood‐ratio tests were not statistically significant for relapse (*χ* ^2^ = 13.48, df = 10; *p* = 0.198) or NRM (*χ* ^2^ = 17.79, df = 10; *p* = 0.059). Individual interaction estimates, particularly for donor subgroups, were imprecise and were therefore considered exploratory; complete results are provided in Table S5.

Abbreviations: ATG, anti‐thymocyte globulin; CR1, first complete remission; fav./interm.‐risk ALL, favourable‐risk ALL, t(12;21)/*ETV6*::*RUNX1* or hyperdiploidy (≥50 chromosomes); HCT‐CI, Hematopoietic Cell Transplantation‐Comorbidity Index; high‐risk ALL, t(9;22)(q34;q11.2) (Philadelphia chromosome, *BCR*::*ABL1*), t(v;11)(q21;q23) (*KMT2A* rearrangement), hypodiploidy (<44 chromosomes), complex karyotype (>3 chromosomal abnormalities) or Ph‐like ALL (Philadelphia chromosome‐like gene expression); disease status at transplantation; intermediate‐risk ALL, patients without high‐risk or favourable abnormalities (e.g. normal karyotype, balanced translocations); MMRD, mismatched related donor; MMUD, mismatched unrelated donor; MSD, matched sibling donor; MUD, matched unrelated donor; NRM, non‐relapse mortality; SDHR, sub‐distribution hazard ratio; TBI, total body irradiation.

Multivariable OS and PFS analyses are shown in Table 4. In the multivariable OS model, 12 Gy TBI was associated with inferior OS (hazard ratio [HR] 2.77, 95% CI 1.15–6.67; *p* = 0.024), as were high‐risk ALL (HR 2.04, 95% CI 1.02–4.09; *p* = 0.043), transplantation beyond CR1 (HR 3.98, 95% CI 2.11–7.50; *p* < 0.001), increasing donor age (HR per year 1.06, 95% CI 1.02–1.09; *p* = 0.001) and higher HCT‐CI score (1–2: HR 2.37, 95% CI 1.02–5.51; *p* = 0.046; ≥3: HR 3.12, 95% CI 1.30–7.49; *p* = 0.011). PFS was associated with high‐risk ALL (HR 2.45, 95% CI 1.26–4.78; *p* = 0.008), transplantation beyond CR1 (HR 3.94, 95% CI 2.12–7.29; *p* < 0.001), HCT‐CI score ≥3 (HR 2.42, 95% CI 1.02–5.73; *p* = 0.044) and increasing donor age (HR per year 1.06, 95% CI 1.03–1.09; *p* < 0.001), but not with TBI dose. Interaction analyses between TBI dose and covariates are shown in Table S6.

**TABLE 4:** Multivariable analyses of overall survival and progression‐free survival.

Variable	Category	OS, HR (95% CI)	*p*‐value	PFS, HR (95% CI)	*p*‐value
TBI dose	8 Gy (ref.)	1.00		1.00	
	12 Gy	2.77 (1.15–6.67)	0.024	2.01 (0.87–4.67)	0.102
Patient age	Per 1‐year increase	1.00 (0.97–1.03)	0.858	1.00 (0.97–1.02)	0.707
Risk group	Fav./interm.‐risk (ref.)	1.00		1.00	
	High‐risk	2.04 (1.02–4.09)	0.043	2.45 (1.26–4.78)	0.008
Disease status	CR1 (ref.)	1.00		1.00	
	>CR1	3.98 (2.11–7.50)	<0.001	3.94 (2.12–7.29)	<0.001
HCT‐CI Score	0 (ref.)	1.00		1.00	
	1–2	2.37 (1.02–5.51)	0.046	2.12 (0.93–4.84)	0.073
	≥3	3.12 (1.30–7.49)	0.011	2.42 (1.02–5.73)	0.044
ATG	No (ref.)	1.00		1.00	
	Yes	1.63 (0.66–4.04)	0.291	1.45 (0.62–3.40)	0.390
Donor type	MSD (ref.)	1.00		1.00	
	MUD	0.59 (0.21–1.62)	0.303	0.52 (0.20–1.33)	0.173
	MMUD, MMRD	1.20 (0.47–3.07)	0.701	0.89 (0.35–2.22)	0.799
	Haploidentical	1.31 (0.32–5.38)	0.710	0.98 (0.25–3.90)	0.982
Donor age	Per 1‐year increase	1.06 (1.02–1.09)	0.001	1.06 (1.03–1.09)	<0.001

*Note*: Exploratory interaction analyses assessed whether the association between TBI dose and outcome varied according to covariates included in the respective multivariable models. Global likelihood‐ratio tests indicated statistically significant interaction between TBI dose and the covariates for OS (*χ* ^2^ = 25.64, df = 10; *p* = 0.004) and PFS (*χ* ^2^ = 19.43, df = 10; *p* = 0.035). Individual interaction estimates, particularly for donor subgroups, were imprecise and were therefore considered exploratory; complete results are provided in Table S6.

Abbreviations: ATG, anti‐thymocyte globulin; CR1, first complete remission; fav./interm.‐risk ALL, favourable‐risk ALL, t(12;21)/*ETV6*::*RUNX1* or hyperdiploidy (≥50 chromosomes); HCT‐CI, Hematopoietic Cell Transplantation‐Comorbidity Index; high‐risk ALL, t(9;22)(q34;q11.2) (Philadelphia chromosome, *BCR*::*ABL1*), t(v;11)(q21;q23) (*KMT2A* rearrangement), hypodiploidy (<44 chromosomes), complex karyotype (>3 chromosomal abnormalities) or Ph‐like ALL (Philadelphia chromosome‐like gene expression); disease status at transplantation; HR, hazard ratio; intermediate‐risk ALL, patients without high‐risk or favourable abnormalities (e.g. normal karyotype, balanced translocations); MMRD, mismatched related donor; MMUD, mismatched unrelated donor; MSD, matched sibling donor; MUD, matched unrelated donor; OS, overall survival; PFS, progression‐free survival; TBI, total body irradiation.

### Sensitivity and exploratory analyses

Sensitivity analyses confirmed the robustness of the association between TBI dose and NRM. In multivariable Fine–Gray models adjusted for transplant year, 12 Gy TBI remained associated with a higher sub‐distribution hazard of NRM (SDHR 5.42, 95% CI 1.48–19.91; *p* = 0.011), with no evidence of an effect on relapse incidence (Table 5). Interaction analyses between TBI dose and covariates are shown in Table S7.

**TABLE 5:** Sensitivity analysis of relapse incidence and non‐relapse mortality using multivariable Fine–Gray regression with additional adjustment for transplant year.

Variable	Category	Relapse, SDHR (95% CI)	*p*‐value	NRM, SDHR (95% CI)	*p*‐value
TBI dose	TBI 8 Gy (ref.)	1.00		1.00	
	TBI 12 Gy	0.53 (0.21–1.34)	0.180	5.42 (1.48–19.91)	0.011
Transplant year	Per 1‐year increase	0.98 (0.92–1.05)	0.570	0.97 (0.88–1.08)	0.600
Patient age	Per 1‐year increase	0.98 (0.95–1.00)	0.100	1.03 (0.98–1.07)	0.240
Risk group	Fav./interm.‐risk (ref.)	1.00		1.00	
	High‐risk	2.52 (1.11–5.70)	0.027	1.44 (0.52–3.98)	0.480
Disease status	CR1 (ref.)	1.00		1.00	
	>CR1	2.63 (1.12–6.16)	0.026	2.45 (0.86–7.01)	0.094
HCT‐CI Score	0 (ref.)	1.00		1.00	
	1–2	1.40 (0.57–3.45)	0.460	2.19 (0.58–8.23)	0.240
	≥3	0.98 (0.28–3.50)	0.980	3.84 (1.00–14.74)	0.050
ATG	No (ref.)	1.00		1.00	
	Yes	0.80 (0.27–2.41)	0.700	1.35 (0.45–4.11)	0.590
Donor type	MSD (ref.)	1.00		1.00	
	MUD	0.43 (0.14–1.35)	0.150	1.44 (0.38–5.42)	0.590
	MMUD, MMRD	0.69 (0.18–2.69)	0.590	2.12 (0.60–7.49)	0.240
	Haploidentical	0.80 (0.16–3.97)	0.780	1.88 (0.12–30.44)	0.660
Donor age	Per 1‐year increase	1.01 (0.98–1.04)	0.610	1.09 (1.03–1.16)	0.003

*Note*: Supplementary analyses were performed to evaluate the robustness of the association between TBI dose and outcomes after adjustment for transplant year. Given its primary relevance for relapse and non‐relapse mortality, adjustment for transplant year was restricted to these end‐points. Exploratory interaction analyses assessed whether the association between TBI dose and outcome varied according to covariates included in the respective multivariable models. Global likelihood‐ratio tests were not statistically significant for relapse (*χ* ^2^ = 14.31, df = 11; *p* = 0.216) or NRM (*χ* ^2^ = 19.54, df = 11; *p* = 0.052). Individual interaction estimates were imprecise, particularly for donor subgroups; complete results are provided in Table S7.

Abbreviations: ATG, anti‐thymocyte globulin; CR1, first complete remission; fav./interm.‐risk ALL, favourable‐risk ALL, t(12;21)/*ETV6*::*RUNX1* or hyperdiploidy (≥50 chromosomes); intermediate‐risk ALL, patients without high‐risk or favourable abnormalities (e.g. normal karyotype, balanced translocations); HCT‐CI, Haematopoietic Cell Transplantation‐Comorbidity Index; high‐risk ALL, t(9;22)(q34;q11.2) (Philadelphia chromosome, *BCR*::*ABL1*), t(v;11)(q21;q23) (*KMT2A* rearrangement), hypodiploidy (<44 chromosomes), complex karyotype (>3 chromosomal abnormalities) or Ph‐like ALL (Philadelphia chromosome‐like gene expression); disease status at transplantation; MMRD, mismatched related donor; MMUD, mismatched unrelated donor; MSD, matched sibling donor; MUD, matched unrelated donor; NRM, non‐relapse mortality; SDHR, sub‐distribution hazard ratio; TBI, total body irradiation.

Consistent findings were observed in cause‐specific Cox models for relapse and NRM. Although no association between 12 Gy TBI and relapse was detected, the association with increased NRM remained stable (CSHR 6.57, 95% CI 1.59–27.03; *p* = 0.009), indicating robustness across modelling approaches (Table S8).

In an exploratory sensitivity analysis additionally adjusted for conditioning regimen category (Table S9), 12 Gy TBI remained associated with a higher sub‐distribution hazard of NRM (SDHR 9.03, 95% CI 1.56–52.18; *p* = 0.014), whereas conditioning regimen category was not associated with NRM. The association between TBI dose and relapse was unchanged.

In the propensity score overlap weighting sensitivity analysis, using age, ALL risk group, disease status, HCT‐CI and transplant year to estimate the propensity score, adequate covariate balance was achieved (all absolute standardized mean differences <0.1; Table S10 and Figure S1). After overlap weighting, 12 Gy TBI remained associated with a higher sub‐distribution hazard of NRM (SDHR 3.46, 95% CI 1.24–9.65; *p* = 0.018), whereas no significant associations were observed for relapse (SDHR 0.52, 95% CI 0.22–1.24; *p* = 0.140), OS (HR 1.20, 95% CI 0.62–2.32; *p* = 0.581) or PFS (HR 1.08, 95% CI 0.56–2.09; *p* = 0.826).

No statistically significant interaction between TBI dose and Philadelphia chromosome status was observed for relapse (interaction SDHR 0.49, 95% CI 0.03–7.69; *p* = 0.610) or NRM (interaction SDHR 2.10, 95% CI 0.12–35.48; *p* = 0.610) (Table S11).

### Secondary outcomes

Detailed multivariable analyses of acute and chronic GVHD are presented in Table S12.

Table S13 summarizes secondary solid malignancies. The 5‐ and 10‐year cumulative incidence of secondary solid malignancies was 3% (95% CI 1–9) and 5% (95% CI 2–11), respectively, with no difference between TBI groups (Gray's test, *p* = 0.200).

## DISCUSSION

This retrospective analysis evaluated the association between TBI dose and outcomes after first allo‐HSCT for adult ALL. Standard‐dose (12 Gy) TBI was associated with higher NRM than reduced‐dose (8 Gy) TBI, without evidence of improved relapse control or PFS. The association with NRM remained consistent across all sensitivity analyses. Although 12 Gy TBI was associated with inferior OS in the multivariable analysis, this association was not observed after propensity score overlap weighting. Thus, the association with OS was not consistent across adjustment approaches.

These findings raise the question of whether the higher NRM associated with standard‐dose TBI is offset by sufficient clinical benefit in adult ALL. Although 12 Gy TBI has historically been used as a standard myeloablative approach, we found no evidence of improved disease control. Our findings are consistent with the European Society for Blood and Marrow Transplantation (EBMT) registry analysis by Spyridonidis et al.,^4^ which likewise observed no improvement in relapse or survival with 12 Gy TBI.

Nearly all patients with Ph + ALL received pre‐transplant TKI therapy, whereas post‐transplant TKI maintenance was uncommon in both TBI dose groups. Evolving TKI strategies may nevertheless have contributed to residual confounding.^2^ Importantly, no significant interaction between TBI dose and Ph status was observed for relapse or NRM, although the wide CIs warrant cautious interpretation.

High‐risk disease and transplantation beyond CR1 were associated with relapse and inferior survival, consistent with previous registry studies.^3^, ^13^ Higher HCT‐CI scores were associated with inferior survival, consistent with the prognostic importance of comorbidity.^14^ Increasing donor age was also associated with higher NRM and inferior survival.^15^, ^16^ Long‐term follow‐up enabled assessment of secondary malignancies. The cumulative incidence of secondary solid malignancies was 5% at 10 years, predominantly non‐melanoma skin cancers, consistent with previous reports.^17^, ^18^

Our findings support continued refinement of conditioning intensity rather than routine escalation of TBI dose. Although prospective randomized data comparing different TBI dose levels are lacking in adults, contemporary paediatric studies emphasize balancing anti‐leukaemic efficacy against treatment‐related toxicity.^19^

Several limitations warrant consideration. The retrospective single‐centre design introduces potential selection bias and residual confounding. Baseline imbalances and confounding by indication may have influenced the results despite multivariable and sensitivity analyses. Differences in TKI use and other treatment practices over time may also have contributed to residual confounding. Consistent pre‐transplant MRD data were unavailable across the study period, limiting adjustment for this major prognostic factor in adult ALL and leaving potential residual confounding. The modest sample size and limited number of events resulted in imprecise multivariable estimates with wide CIs, particularly for subgroup and interaction analyses. Finally, changes in supportive care, donor selection and GVHD prophylaxis over the study period may have influenced outcomes despite adjustment.

Compared with 8 Gy TBI, standard‐dose (12 Gy) TBI was associated with increased NRM without evidence of improved leukaemia control in adults undergoing first allo‐HSCT for ALL. Although 12 Gy TBI was associated with inferior OS in the multivariable analysis, this finding was not confirmed after propensity score overlap weighting. These findings support prospective evaluation of reduced‐dose TBI strategies in appropriately selected patients. Future studies should define which patients benefit from reduced‐dose TBI while optimizing disease control through risk stratification and post‐transplant strategies.

## AUTHOR CONTRIBUTIONS

**Isabella Gruber:** Project administration; conceptualization; investigation; formal analysis; visualization; methodology; writing – original draft; writing – review and editing. **Matthias Edinger:** Data curation; resources; writing – review and editing. **Hendrik Poeck:** Resources; data curation; writing – review and editing. **Elisabeth Meedt:** Data curation. **Ernst Holler:** Resources; data curation; writing – review and editing. **Wolfgang Herr:** Supervision; resources; writing – review and editing. **Oliver Koelbl:** Resources; writing – review and editing; supervision. **Daniel Wolff:** Resources; supervision; conceptualization; data curation; validation; writing – review and editing.

## FUNDING INFORMATION

No specific funding was received for this study.

## CONFLICT OF INTEREST STATEMENT

Daniel Wolff received Research Grant from Novartis and honoraria from Novartis, Incyte, Sanofi, Behring, Neovii and Therakos, all outside the submitted work. The remaining authors declare no competing interests.

## ETHICS STATEMENT

This retrospective chart review study involving human participants was in accordance with the ethical standards of the institutional and national research committee and with the 1964 Helsinki Declaration and its later amendments or comparable ethical standards. The study was approved by the Ethics Committee of the University of Regensburg (reference number 20‐1810‐101; approval date: 22 April 2020).

## PATIENT CONSENT STATEMENT

Informed consent was obtained from all individual participants included in the study.

## CLINICAL TRIAL REGISTRATION

This retrospective observational study was not registered as a clinical trial.

## Supporting information


**Table S1.** TBI‐based conditioning regimens before first allogeneic haematopoietic stem cell transplantation in adults with acute lymphoblastic leukaemia.
**Table S2.** Tyrosine kinase inhibitor (TKI) therapy among patients with Philadelphia chromosome‐positive acute lymphoblastic leukaemia according to TBI dose.
**Table S3.** Engraftment and donor chimerism after allo‐HSCT.
**Table S4.** Causes of death.
**Table S5.** Interaction analyses between TBI dose and covariates included in the multivariable Fine–Gray models for relapse and non‐relapse mortality.
**Table S6.** Interaction analyses between TBI dose and covariates included in the multivariable Cox regression models for overall survival and progression‐free survival.
**Table S7.** Interaction analyses between TBI dose and covariates included in the multivariable Fine–Gray models for relapse and non‐relapse mortality with additional adjustment for transplant year.
**Table S8.** Sensitivity analysis of relapse incidence and non‐relapse mortality using multivariable cause‐specific Cox regression with additional adjustment for transplant year.
**Table S9.** Exploratory sensitivity analysis adjusting for conditioning regimen category in multivariable Fine–Gray models for relapse incidence and non‐relapse mortality.
**Table S10.** Covariate balance before and after propensity score overlap weighting.
**Figure S1.** Covariate balance before and after propensity score overlap weighting. Absolute standardized mean differences for all covariates before and after overlap weighting. The dashed line indicates the prespecified threshold of 0.1 for adequate covariate balance.
**Table S11.** Interaction term between TBI dose and Philadelphia chromosome status.
**Table S12.** Multivariable Fine–Gray regression analyses of acute and chronic graft‐versus‐host disease.
**Table S13.** Characteristics of secondary solid malignancies following TBI‐based allogeneic haematopoietic stem cell transplantation.

## Data Availability

The data that support the findings of this study are available on request from the corresponding author. The data are not publicly available due to privacy or ethical restrictions.
