# Supplementary material for: Reduced‐dose versus standard‐dose total body irradiation before allogeneic haematopoietic stem cell transplantation in adults with acute lymphoblastic leukaemia
Source: Br J Haematol. 2026 Sep 15;209(5):2203–12. doi: 10.1111/bjh.70835 (PMC13748353; doi:10.1111/bjh.70835)
Supplement: Supplementary file 1 [file BJH-209-2203-s001.docx]

**Supplementary Table S1** TBI-based conditioning regimens before first allogeneic haematopoietic stem cell transplantation in adults with acute lymphoblastic leukaemia.

| **Regimen** | **Description** | **n** | **%** |
| --- | --- | --- | --- |
| Cy2/Flu/TBI 8 Gy | Cyclophosphamide 2 × 60 mg/kg, fludarabine 3 × 30 mg/m², TBI 8 Gy (4 × 2 Gy) | 15 | 14.9% |
| Flu/TBI 8 Gy | Fludarabine 4 × 30 mg/m², TBI 8 Gy (4 × 2 Gy) | 33 | 32.7% |
| Flu/TBI 12 Gy | Fludarabine 4 × 30 mg/m², TBI 12 Gy (6 × 2 Gy) | 4 | 4.0% |
| VP16/TBI 8 Gy | VP16 1 × 25–40 mg/kg, TBI 8 Gy (4 × 2 Gy) | 2 | 2.0% |
| Cy2/TBI 8 Gy | Cyclophosphamide 2 × 60 mg/kg, TBI 8 Gy (4 × 2 Gy) | 1 | 1.0% |
| Cy2/TBI 12 Gy | Cyclophosphamide 2 × 60 mg/kg, TBI 12 Gy (6 × 2 Gy) | 37 | 36.6% |
| VP16/TBI 12 Gy | VP16 1 × 30–60 mg/kg, TBI 12 Gy (6 × 2 Gy) | 9 | 8.9% |
| **Table footnote**:  VP16 was administered according to mucositis risk.  **Abbreviations**:  TBI, total body irradiation; VP16, etoposide. | | | |

**Supplementary Table S2** Tyrosine kinase inhibitor (TKI) therapy among patients with Philadelphia chromosome-positive acute lymphoblastic leukaemia according to TBI dose.

| **Ph+ ALL** | **TBI 8 Gy**  **(n = 19)** | **TBI 12 Gy**  **(n = 10)** |
| --- | --- | --- |
| Pre-HSCT TKI therapy | 19/19 (100.0%) | 9/10 (90.0%) |
| Post-HSCT TKI therapy | 4/19 (21.1%) | 2/10 (20.0%) |
| **Abbreviations**:  HSCT, haematopoietic stem cell transplantation; TBI, total body irradiation; TKI, tyrosine kinase inhibitor. | | |

**Supplementary Table S3** Engraftment and donor chimerism after allo-HSCT.

| **Outcome** | **TBI 8 Gy**  **(n = 51)** | **TBI 12 Gy**  **(n = 50)** | ***p*-value** |
| --- | --- | --- | --- |
| Time to neutrophil recovery, days, median (IQR) | 18 (15–21) | 17 (13–21) | 0.673 |
| Time to platelet recovery, days, median (IQR) | 19 (13–24) | 18 (15–24) | 0.833 |
| Full donor chimerism by day 28, n/N (%) | 46/50 (92.0%) | 46/47 (97.9%) | 0.363 |
| Primary graft failure, n | 1 | 0 | - |
| Secondary graft failure, n | 0 | 1 | - |
| **Table footnote**:  Neutrophil recovery was defined as ANC >500 cells/μL and platelet recovery as platelet count ≥20,000/μL. Donor chimerism was assessed at day 28; patients with graft failure or death before day 28 were not evaluable.  **Abbreviations**:  HSCT, haematopoietic stem cell transplantation; TBI, total body irradiation; ANC, absolute neutrophil count; IQR, interquartile range. | | | |

**Supplementary Table S4** Causes of death.

|  |  | **All**  **(n = 48)** | **TBI 8 Gy**  **(n = 24)** | **TBI 12 Gy**  **(n = 24)** | ***p*-value** |
| --- | --- | --- | --- | --- | --- |
| Causes of death | Relapse of ALL | 24 (50.0%) | 14 (58.3%) | 10 (41.7%) | 0.387 |
|  | NRM | 24 (50.0%) | 10 (41.7%) | 14 (58.3%) |  |
| **Table footnote**:  Among patients experiencing NRM, 14 deaths (58%) were attributed to graft-versus-host disease (GVHD), 7 (29%) to infections (including bacterial, viral, fungal, parasitic, mixed, or unknown), and 3 (13%) to other causes (1 secondary solid malignancy, 1 myocardial infarction, and 1 neurodegeneration). These subcategories were analysed descriptively without formal statistical comparison.  **Abbreviations**:  TBI, total body irradiation; NRM, non-relapse mortality; ALL, acute lymphoblastic leukaemia. | | | | | |

**Supplementary Table S5** Interaction analyses between TBI dose and covariates included in the multivariable Fine–Gray models for relapse and non-relapse mortality.

| **Covariate interaction**  **term** | **Relapse, SDHR (95% CI)** | ***p*-value** | **NRM, SDHR (95% CI)** | ***p*-value** |
| --- | --- | --- | --- | --- |
| Patient age | 1.08 (0.94–1.23) | 0.290 | 0.95 (0.86–1.05) | 0.280 |
| High-risk ALL | 1.76 (0.32–9.82) | 0.520 | 11.59 (0.73–185.13) | 0.083 |
| Disease status >CR1 | 1.54 (0.24–9.66) | 0.650 | 3.72 (0.19–73.41) | 0.390 |
| HCT-CI 1–2 | 1.38 (0.24–8.04) | 0.720 | 5.16 (0.06–478.29) | 0.480 |
| HCT-CI ≥3 | 4.04 (0.39–42.17) | 0.240 | 1.27 (0.02–97.42) | 0.920 |
| ATG | 0.12 (0.02–0.99) | 0.049 | 16.79 (0.39–725.16) | 0.140 |
| MUD | 4.53 (0.40–51.57) | 0.220 | 0.38 (0.02–6.78) | 0.510 |
| MMUD, MMRD | 22.61 (1.45–352.09) | 0.026 | 0.33 (0.03–3.82) | 0.370 |
| Haploidentical donor | 3.69 × 10⁻⁵ (1.95×10⁻⁶–6.97×10⁻⁴) | <0.001 | 5.41 × 10⁶ (2.67×10⁵–1.10×10⁸) | <0.001 |
| Donor age | 0.95 (0.87–1.05) | 0.310 | 1.01 (0.87–1.17) | 0.900 |
| **Table footnote**:  Global interaction test: NRM, likelihood-ratio χ² = 17.79, df = 10, *p* = 0.059; relapse, likelihood-ratio χ² = 13.48, df = 10, *p* = 0.198.  The interaction estimate for the haploidentical donor subgroup was highly unstable because of the limited number of patients in this subgroup and should therefore be interpreted with caution.  **Reference categories:**  favourable/intermediate-risk ALL for high-risk ALL; CR1 for disease status; HCT-CI 0 for HCT-CI 1–2 and ≥3; no ATG for ATG; MSD for MUD, MMUD/MMRD, and haploidentical donor.  **Abbreviations**:  NRM, non-relapse mortality; SDHR, sub‑distribution hazard ratio; TBI, total body irradiation; high-risk ALL, t(9;22)(q34;q11.2) (Philadelphia chromosome, *BCR*::*ABL1*), t(v;11)(q21;q23) (*KMT2A* rearrangement), hypodiploidy (<44 chromosomes), complex karyotype (>3 chromosomal abnormalities), or Ph-like ALL (Philadelphia chromosome-like gene expression); favourable/intermediate-risk ALL, favourable-risk ALL, t(12;21)/*ETV6*::*RUNX1* or hyperdiploidy (≥50 chromosomes); intermediate-risk ALL, patients without high-risk or favourable abnormalities (e.g., normal karyotype, balanced translocations); disease status at transplantation, CR1, first complete remission; HCT-CI, Hematopoietic Cell Transplantation‑Comorbidity Index; ATG, anti‑thymocyte globulin; MSD, matched sibling donor; MUD, matched unrelated donor; MMUD, mismatched unrelated donor; MMRD, mismatched related donor. | | | | |

**Supplementary Table S6** Interaction analyses between TBI dose and covariates included in the multivariable Cox regression models for overall survival and progression-free survival.

| **Covariate interaction**  **term** | **OS, HR (95% CI)** | ***p*-value** | **PFS, HR (95% CI)** | ***p*-value** |
| --- | --- | --- | --- | --- |
| Patient age | 0.99 (0.92–1.07) | 0.829 | 1.02 (0.95–1.09) | 0.666 |
| High-risk ALL | 22.64 (3.39–151.04) | 0.001 | 7.71 (1.43–41.47) | 0.017 |
| Disease status >CR1 | 13.11 (1.85–92.85) | 0.010 | 4.88 (0.89–26.84) | 0.068 |
| HCT-CI 1–2 | 4.30 (0.66–28.03) | 0.128 | 4.84 (0.84–28.04) | 0.078 |
| HCT-CI ≥3 | 3.41 (0.53–21.81) | 0.194 | 4.45 (0.75–26.25) | 0.099 |
| ATG | 2.64 (0.39–17.91) | 0.321 | 0.99 (0.18–5.48) | 0.988 |
| MUD | 3.15 (0.42–23.54) | 0.264 | 4.71 (0.65–34.04) | 0.125 |
| MMUD, MMRD | 9.93 (1.41–69.95) | 0.021 | 12.41 (1.69–91.04) | 0.013 |
| Haploidentical donor | 153.33 (6.32–3720.89) | 0.002 | 43.44 (2.32–812.78) | 0.012 |
| Donor age | 1.04 (0.97–1.12) | 0.243 | 1.00 (0.93–1.07) | 0.963 |
| **Table footnote**:  Global interaction test: OS, likelihood-ratio χ² = 25.64, df = 10, *p* = 0.004; PFS, likelihood-ratio χ² = 19.43, df = 10, *p* = 0.035.  The interaction estimate for the haploidentical donor subgroup was highly unstable because of the limited number of patients in this subgroup and should therefore be interpreted with caution.  **Reference categories:**  favourable/intermediate-risk ALL for high-risk ALL; CR1 for disease status; HCT-CI 0 for HCT-CI 1–2 and ≥3; no ATG for ATG; MSD for MUD, MMUD/MMRD, and haploidentical donor.  **Abbreviations**:  OS, overall survival; PFS, progression-free survival; NRM, non-relapse mortality; HR, hazard ratio; TBI, total body irradiation; high-risk ALL, t(9;22)(q34;q11.2) (Philadelphia chromosome, *BCR*::*ABL1*), t(v;11)(q21;q23) (*KMT2A* rearrangement), hypodiploidy (<44 chromosomes), complex karyotype (>3 chromosomal abnormalities), or Ph‑like ALL (Philadelphia chromosome-like gene expression); favourable/intermediate-risk ALL, favourable-risk ALL, t(12;21)/*ETV6*::*RUNX1* or hyperdiploidy (≥50 chromosomes); intermediate-risk ALL, patients without high-risk or favourable abnormalities (e.g., normal karyotype, balanced translocations); disease status at transplantation, CR1, first complete remission; HCT-CI, Hematopoietic Cell Transplantation‑Comorbidity Index; ATG, anti‑thymocyte globulin; MSD, matched sibling donor; MUD, matched unrelated donor; MMUD, mismatched unrelated donor; MMRD, mismatched related donor. | | | | |

**Supplementary Table S7** Interaction analyses between TBI dose and covariates included in the multivariable Fine–Gray models for relapse and non-relapse mortality with additional adjustment for transplant year.

| **Covariate interaction**  **term** | **Relapse, SDHR (95% CI)** | ***p*‑value** | **NRM, SDHR (95% CI)** | ***p*‑value** |
| --- | --- | --- | --- | --- |
| Transplant year | 1.08 (0.93–1.26) | 0.290 | 0.92 (0.74–1.14) | 0.430 |
| Patient age | 1.07 (0.94–1.22) | 0.330 | 0.95 (0.85–1.06) | 0.330 |
| High-risk ALL | 1.90 (0.36–10.04) | 0.450 | 15.11 (1.02–222.72) | 0.048 |
| Disease status >CR1 | 2.66 (0.27–26.12) | 0.400 | 3.93 (0.14–112.10) | 0.420 |
| HCT-CI 1–2 | 1.24 (0.24–6.42) | 0.800 | 4.61 (0.05–434.25) | 0.510 |
| HCT-CI ≥3 | 4.63 (0.45–48.05) | 0.200 | 0.73 (0.01– 58.26) | 0.890 |
| ATG | 0.13 (0.02–0.98) | 0.047 | 11.75 (0.26–537.44) | 0.210 |
| MUD | 4.14 (0.35–48.98) | 0.260 | 0.63 (0.03–13.63) | 0.770 |
| MMUD, MMRD | 19.23 (1.40–264.92) | 0.027 | 0.60 (0.05–7.93) | 0.700 |
| Haploidentical donor | 1.86 × 10⁻⁵ (7.76×10⁻⁷–4.45×10⁻⁴) | <0.001 | 1.95 × 10⁷ (2.30×10⁵–1.66×10⁹) | <0.001 |
| Donor age | 0.97 (0.89–1.05) | 0.430 | 0.98 (0.85–1.13) | 0.770 |
| **Table footnote**:  Global interaction test: NRM, likelihood-ratio χ² = 19.54, df = 11, *p* = 0.052; relapse, likelihood-ratio χ² = 14.31, df = 11, *p =* 0.216.  The interaction estimate for the haploidentical donor subgroup was highly unstable because of the limited number of patients in this subgroup and should therefore be interpreted with caution.  **Reference categories:**  favourable/intermediate-risk ALL for high-risk ALL; CR1 for disease status; HCT-CI 0 for HCT-CI 1–2 and ≥3; no ATG for ATG; MSD for MUD, MMUD/MMRD, and haploidentical donor.  **Abbreviations**:  NRM, non-relapse mortality; SDHR, sub‑distribution hazard ratio; TBI, total body irradiation; high-risk ALL, t(9;22)(q34;q11.2) (Philadelphia chromosome, *BCR*::*ABL1*), t(v;11)(q21;q23) (*KMT2A* rearrangement), hypodiploidy (<44 chromosomes), complex karyotype (>3 chromosomal abnormalities), or Ph-like ALL (Philadelphia chromosome-like gene expression); favourable/intermediate-risk ALL, favourable-risk ALL, t(12;21)/*ETV6*::*RUNX1* or hyperdiploidy (≥50 chromosomes); intermediate-risk ALL, patients without high-risk or favourable abnormalities (e.g., normal karyotype, balanced translocations); disease status at transplantation, CR1, first complete remission; HCT-CI, Hematopoietic Cell Transplantation‑Comorbidity Index; ATG, anti‑thymocyte globulin; MSD, matched sibling donor; MUD, matched unrelated donor; MMUD, mismatched unrelated donor; MMRD, mismatched related donor. | | | | |

**Supplementary Table S8** Sensitivity analysis of relapse incidence and non-relapse mortality using multivariable cause-specific Cox regression with additional adjustment for transplant year.

| **Variable** | **Category** | **Relapse, CSHR (95% CI)** | ***p*‑value** | **NRM, CSHR (95% CI)** | ***p*-value** |
| --- | --- | --- | --- | --- | --- |
| TBI dose | TBI 8 Gy (reference) | 1.00 |  | 1.00 |  |
|  | TBI 12 Gy | 0.79 (0.28−2.24) | 0.662 | 6.57 (1.59–27.03) | 0.009 |
| Transplant year | per 1-year increase | 0.98 (0.91−1.05) | 0.582 | 0.99 (0.90–1.09) | 0.830 |
| Patient age | per 1-year increase | 0.98 (0.95−1.02) | 0.337 | 1.02 (0.98−1.07) | 0.364 |
| Risk group | Fav./interm.-risk (ref.) | 1.00 |  | 1.00 |  |
|  | High-risk | 2.82 (1.10−7.19) | 0.030 | 2.28 (0.86−6.01) | 0.096 |
| Disease status | CR1 (ref.) | 1.00 |  | 1.00 |  |
|  | >CR1 | 3.49 (1.35−9.00) | 0.010 | 4.35 (1.50−12.63) | 0.007 |
| HCT-CI Score | 0 (ref.) | 1.00 |  | 1.00 |  |
|  | 1–2 | 1.64 (0.60−4.47) | 0.335 | 3.11 (0.86−11.26) | 0.083 |
|  | ≥3 | 1.22 (0.38−3.89) | 0.741 | 4.71 (1.21−18.30) | 0.025 |
| ATG | No (ref.) | 1.00 |  | 1.00 |  |
|  | Yes | 0.94 (0.31−2.88) | 0.919 | 1.53 (0.39−6.00) | 0.541 |
| Donor type | MSD (ref.) | 1.00 |  | 1.00 |  |
|  | MUD | 0.37 (0.11−1.25) | 0.110 | 1.05 (0.22−4.93) | 0.948 |
|  | MMUD, MMRD | 0.64 (0.18−2.23) | 0.480 | 2.08 (0.45−9.62) | 0.349 |
|  | Haploidentical | 0.83 (0.14−4.87) | 0.837 | 1.51 (0.12−18.96) | 0.751 |
| Donor age | per 1-year increase | 1.03 (0.99−1.07) | 0.203 | 1.10 (1.04−1.16) | <0.001 |
| **Table footnote**:  Supplementary analyses were performed to evaluate the robustness of the association between TBI dose and outcomes after adjustment for transplant year. Given its primary relevance for relapse and non-relapse mortality, adjustment for transplant year was restricted to these endpoints.  **Abbreviations**:  CSHR, cause-specific hazard ratio estimated using cause-specific Cox proportional hazards regression, with competing events censored; TBI, total body irradiation; fav./interm.-risk ALL, favourable-risk ALL, t(12;21)/*ETV6*::*RUNX1* or hyperdiploidy (≥50 chromosomes); intermediate-risk ALL, patients without high-risk or favourable abnormalities (e.g., normal karyotype, balanced translocations); high-risk ALL, t(9;22)(q34;q11.2) (Philadelphia chromosome, *BCR*::*ABL1*), t(v;11)(q21;q23) (*KMT2A* rearrangement), hypodiploidy (<44 chromosomes), complex karyotype (>3 chromosomal abnormalities), or Ph‑like ALL (Philadelphia chromosome-like gene expression); disease status at transplantation, CR1, first complete remission; HCT-CI, Hematopoietic Cell Transplantation‑Comorbidity Index; ATG, anti‑thymocyte globulin; MSD, matched sibling donor; MUD, matched unrelated donor; MMUD, mismatched unrelated donor; MMRD, mismatched related donor. | | | | | |

**Supplementary Table S9** Exploratory sensitivity analysis adjusting for conditioning regimen category in multivariable Fine–Gray models for relapse incidence and non-relapse mortality.

| **Variable** | **Category** | **Relapse, SDHR (95% CI)** | ***p-*value** | **NRM, SDHR (95% CI)** | ***p*-value** |
| --- | --- | --- | --- | --- | --- |
| TBI dose | 8 Gy (ref.) | 1.00 |  | 1.00 |  |
|  | 12 Gy | 0.35 (0.09–1.37) | 0.130 | 9.03 (1.56–52.18) | 0.014 |
| Conditioning regimen | Flu-containing (ref.) | 1.00 |  | 1.00 |  |
|  | Cy/VP16-containing | 1.71 (0.41–7.21) | 0.460 | 0.64 (0.18–2.29) | 0.500 |
| Patient age | per 1-year increase | 0.98 (0.95–1.01) | 0.120 | 1.02 (0.98–1.06) | 0.330 |
| Risk group | Fav./interm.-risk (ref.) | 1.00 |  | 1.00 |  |
|  | High-risk | 2.43 (1.04–5.71) | 0.041 | 1.52 (0.58–3.96) | 0.390 |
| Disease status | CR1 (ref.) | 1.00 |  | 1.00 |  |
|  | >CR1 | 3.12 (1.46–6.67) | 0.003 | 2.82 (1.21–6.57) | 0.017 |
| HCT-CI Score | 0 (ref.) | 1.00 |  | 1.00 |  |
|  | 1–2 | 1.44 (0.58–3.59) | 0.430 | 2.23 (0.59–8.45) | 0.240 |
|  | ≥3 | 1.10 (0.31–3.93) | 0.880 | 4.19 (1.14–15.43) | 0.031 |
| ATG | No (ref.) | 1.00 |  | 1.00 |  |
|  | Yes | 0.79 (0.27–2.35) | 0.670 | 1.48 (0.46–4.69) | 0.510 |
| Donor type | MSD (ref.) | 1.00 |  | 1.00 |  |
|  | MUD | 0.46 (0.14–1.55) | 0.210 | 1.36 (0.36–5.16) | 0.650 |
|  | MMUD, MMRD | 0.66 (0.17–2.59) | 0.550 | 1.94 (0.54–6.96) | 0.310 |
|  | Haploidentical | 0.63 (0.15–2.75) | 0.540 | 1.69 (0.10–29.23) | 0.720 |
| Donor age | per 1-year increase | 1.01 (0.98–1.04) | 0.570 | 1.10 (1.05–1.16) | <0.001 |
| **Table footnote**:  An exploratory sensitivity analysis was performed by additionally including conditioning chemotherapy category as a covariate in the prespecified multivariable Fine–Gray models. Conditioning regimens were categorized as Flu-containing (fludarabine/TBI or cyclophosphamide/fludarabine/TBI) and Cy/VP16-containing (cyclophosphamide/TBI or etoposide [VP16]/TBI) regimens.  **Abbreviations**:  SDHR, sub-distribution hazard ratio; fav./interm.-risk ALL, favourable-risk ALL, t(12;21)/*ETV6*::*RUNX1* or hyperdiploidy (≥50 chromosomes); intermediate-risk ALL, patients without high-risk or favourable abnormalities (e.g., normal karyotype, balanced translocations); high-risk ALL, t(9;22)(q34;q11.2) (Philadelphia chromosome, *BCR*::*ABL1*), t(v;11)(q21;q23) (*KMT2A* rearrangement), hypodiploidy (<44 chromosomes), complex karyotype (>3 chromosomal abnormalities), or Ph‑like ALL (Philadelphia chromosome-like gene expression); disease status at transplantation, CR1, first complete remission; HCT-CI, Hematopoietic Cell Transplantation‑Comorbidity Index; MSD, matched sibling donor; MUD, matched unrelated donor; MMUD, mismatched unrelated donor; MMRD, mismatched related donor. | | | | | |

**Supplementary Table S10** Covariate balance before and after propensity score overlap weighting.

| **Covariate** | **Before ATO weighting (SMD)** | **After ATO weighting (SMD)** |
| --- | --- | --- |
| Patient age | 1.58 | 0.00 |
| ALL risk group: high-risk | 0.60 | 0.00 |
| Treatment year | 0.45 | 0.00 |
| HCT-CI ≥3 | 0.40 | 0.00 |
| HCT-CI 1–2 | 0.36 | 0.00 |
| Disease status: >CR1 | 0.18 | 0.00 |
| **Table footnote**:  Standardized mean differences are presented before and after propensity score overlap weighting. Values are presented as absolute SMDs and rounded to two decimal places. Absolute SMDs <0.1 were considered indicative of adequate covariate balance.  **Abbreviations**:  ATO, average treatment effect in the overlap population; SMD, standardized mean difference; high-risk ALL, t(9;22)(q34;q11.2) (Philadelphia chromosome, *BCR*::*ABL1*), t(v;11)(q21;q23) (*KMT2A* rearrangement), hypodiploidy (<44 chromosomes), complex karyotype (>3 chromosomal abnormalities), or Ph-like ALL (Philadelphia chromosome-like gene expression); disease status at transplantation, CR1, first complete remission; HCT-CI, Hematopoietic Cell Transplantation‑Comorbidity Index. | | |

**Supplementary Figure S1** Covariate balance before and after propensity score overlap weighting. Absolute standardized mean differences for all covariates before and after overlap weighting. The dashed line indicates the prespecified threshold of 0.1 for adequate covariate balance.

**Supplementary Table S11** Interaction term between TBI dose and Philadelphia chromosome status.

| **Outcome** | **TBI dose x Ph status, HR** | **95% CI** | ***p*-value** |
| --- | --- | --- | --- |
| Relapse | 0.49 | 0.03–7.69 | 0.610 |
| NRM | 2.10 | 0.12–35.48 | 0.610 |
| **Abbreviations**:  NRM, non-relapse mortality; HRs (hazard ratios) represent the interaction term between TBI dose (12 Gy vs. 8 Gy) and Philadelphia chromosome status (Ph+ vs. Ph−); CI, confidence interval. | | | |

**Supplementary Table S12** Multivariable Fine–Gray regression analyses of acute and chronic graft‑versus-host disease.

| **Variable** | **Category** | **aGVHD, SDHR (95% CI)** | ***p*‑value** | **cGVHD, SDHR (95% CI)** | ***p*‑value** |
| --- | --- | --- | --- | --- | --- |
| TBI dose | 8 Gy (ref.) | 1.00 |  | 1.00 |  |
|  | 12 Gy | 1.40 (0.66−3.00) | 0.380 | 0.83 (0.36−1.90) | 0.660 |
| Patient age | per 1-year increase | 1.01 (0.99−1.04) | 0.300 | 1.01 (0.97−1.04) | 0.700 |
| Risk group | Fav./interm.-risk (ref.) | 1.00 |  | 1.00 |  |
|  | High-risk | 0.91 (0.48−1.71) | 0.760 | 1.18 (0.56−2.51) | 0.660 |
| Disease status | CR1 (ref.) | 1.00 |  | 1.00 |  |
|  | >CR1 | 1.35 (0.73−2.48) | 0.340 | 1.17 (0.54−2.52) | 0.700 |
| HCT-CI Score | 0 (ref.) | 1.00 |  | 1.00 |  |
|  | 1–2 | 1.32 (0.63−2.78) | 0.460 | 0.83 (0.40−1.75) | 0.630 |
|  | ≥3 | 1.09 (0.44−2.68) | 0.850 | 1.00 (0.44−2.30) | 1.00 |
| ATG | No (ref.) | 1.00 |  | 1.00 |  |
|  | Yes | 1.10 (0.47−2.60) | 0.820 | 0.35 (0.14−0.86) | 0.022 |
| GVHD prophylaxis | CyA/MTX (ref.) | 1.00 |  | 1.00 |  |
|  | CyA/MMF, Tac/MMF | 0.94 (0.37−2.38) | 0.900 | 1.97 (0.71−5.48) | 0.190 |
|  | PT-Cy/Tac/MMF | 0.79 (0.19−3.27) | 0.750 | 0.21 (0.04−1.03) | 0.054 |
| Donor type | MSD (ref.) | 1.00 |  | 1.00 |  |
|  | MUD | 1.29 (0.51−3.28) | 0.600 | 1.84 (0.66−5.17) | 0.240 |
|  | MMUD, MMRD | 1.05 (0.39−2.79) | 0.920 | 1.35 (0.45−4.06) | 0.590 |
|  | Haploidentical | 1.64 (0.33−8.16) | 0.550 | 3.91 (0.67−22.8) | 0.130 |
| Donor age | per 1-year increase | 1.00 (0.97−1.02) | 0.790 | 0.98 (0.96−1.01) | 0.250 |
| **Abbreviations**:  aGVHD, acute graft-versus-host disease; cGVHD, chronic graft-versus-host disease; SDHR, sub-distribution hazard ratio; fav./interm.-risk ALL, favourable-risk ALL, t(12;21)/*ETV6*::*RUNX1* or hyperdiploidy (≥50 chromosomes); intermediate-risk ALL, patients without high-risk or favourable abnormalities (e.g., normal karyotype, balanced translocations); high-risk ALL, t(9;22)(q34;q11.2) (Philadelphia chromosome, *BCR*::*ABL1*), t(v;11)(q21;q23) (*KMT2A* rearrangement), hypodiploidy (<44 chromosomes), complex karyotype (>3 chromosomal abnormalities), or Ph-like ALL (Philadelphia chromosome-like gene expression); disease status at transplantation, CR1, first complete remission; HCT-CI, Hematopoietic Cell Transplantation‑Comorbidity Index; CyA/MTX, cyclosporine and methotrexate; CyA/MMF, cyclosporine and mycophenolate mofetil; Tac/MMF, tacrolimus and mycophenolate mofetil; post-transplant Cy/Tac/MMF, post-transplant cyclophosphamide, tacrolimus, and mycophenolate mofetil (primarily used in haploidentical transplantation); MSD, matched sibling donor; MUD, matched unrelated donor; MMUD, mismatched unrelated donor; MMRD, mismatched related donor. | | | | | |

**Supplementary Table S13** Characteristics of secondary solid malignancies following TBI-based allogeneic haematopoietic stem cell transplantation.

| **Pat.**  **No** | **Type of secondary solid malignancy** | **Conditioning regimen** | **Age at HSCT** | **Sex** | **Years from HSCT to SSM** | **GVHD prophylaxis** | **Acute**  **GVHD** | **Chronic GVHD requiring systemic IS, maximum staging** |
| --- | --- | --- | --- | --- | --- | --- | --- | --- |
| Squamous cell carcinoma (n = 8) in 3 patients | | | | | | | | |
| 43 | Squamous cell carcinoma, ear, pT2 cN0 | Flu/Cy/TBI 8 Gy | 51.3 | Male | 11.9 | CyA/MTX, ATG | None | None |
| 43 | Squamous cell carcinoma, face, pT1 cN0 | Flu/Cy/TBI 8 Gy | 51.3 | Male | 14.1 | CyA/MTX, ATG | None | None |
| 52 | Squamous cell carcinoma, lip, pT1 cN0 | Flu/TBI 8 Gy | 56.4 | Male | 3.7 | CyA/MTX, ATG | None | Skin 2, Eyes 1, Oral mucosa 1, ITP |
| 52 | Squamous cell carcinoma, scalp, pT1cN0 | Flu/TBI 8 Gy | 56.4 | Male | 3.7 | CyA/MTX, ATG | None | Skin 2, Eyes 1, Oral mucosa 1, ITP |
| 59 | Squamous cell carcinoma, face, pT1 cN0 | Flu/TBI 8 Gy | 64.0 | Male | 1.9 | PT-Cy/Tacro/MMF | Skin 2 | Skin 2 |
| 59 | Squamous cell carcinoma, forearm | Flu/TBI 8 Gy | 64.0 | Male | 6.9 | PT-Cy/Tacro/MMF | Skin 2 | Skin 2 |
| 59 | Squamous cell carcinoma, front | Flu/TBI 8 Gy | 64.0 | Male | 7.3 | PT-Cy/Tacro/MMF | Skin 2 | Skin 2 |
| 59 | Squamous cell carcinoma, cheek | Flu/TBI 8 Gy | 64.0 | Male | 7.3 | PT-Cy/Tacro/MMF | Skin 2 | Skin 2 |
| Non-squamous cell carcinoma (n = 11) in 7 patients | | | | | | | | |
| 21 | Grade 2 pancreatic neuroendocrine tumour, pM1a | Cy2/TBI 12 Gy | 39.0 | Male | 10.2 † | CyA/MTX, ATG | Skin 1 | None |
| 37 | Atypical meningioma, WHO grade 2 | Cy2/TBI 12 Gy | 18.0 | Male | 15.0 | CyA/MTX, ATG | Skin 2 | Skin 3, Fascia 3, Oral 2 |
| 43 | Basal cell carcinoma, forearm, pT1 cN0 | Flu/Cy/TBI 8 Gy | 51.3 | Male | 6.5 | CyA/MTX, ATG | None | None |
| 43 | Basal cell carcinoma, scalp, pT1 cN0 | Flu/Cy/TBI 8 Gy | 51.3 | Male | 11.7 | CyA/MTX, ATG | None | None |
| 43 | Basal cell carcinoma, nose, pT2 cN0 | Flu/Cy/TBI 8 Gy | 51.3 | Male | 13.8 | CyA/MTX, ATG | None | None |
| 43 | Basal cell carcinoma, face, pT1 cN0 | Flu/Cy/TBI 8 Gy | 51.3 | Male | 14.2 | CyA/MTX, ATG | None | None |
| 49 | Pleomorphic dermal sarcoma, scalp, pT2 cN0 cM0 | Flu/TBI 8 Gy | 69.0 | Male | 11.4 | CyA/MTX, ATG | None | Polyserositis 2, Skin 1, Intestine 1 |
| 52 | Basal cell carcinoma, face | Flu/TBI 8 Gy | 56.4 | Male | 6.0 | CyA/MTX, ATG | None | Skin 2, Eyes 1, Oral mucosa 1, ITP |
| 59 | Basal cell carcinoma, shoulder | Flu/TBI 8 Gy | 64.0 | Male | 1.7 | PT-Cy/Tacro/MMF | Skin 2 | Skin 2 |
| 59 | Malignant melanoma, scalp, pT4b cN0 pM1b | Flu/TBI 8 Gy | 64.0 | Male | 4.7 | PT-Cy/Tacro/MMF | Skin 2 | Skin 2 |
| 84 | Malignant melanoma, knee, pT1 cN0 | Flu/TBI 12 Gy | 34.0 | Male | 2.1 | CyA/MTX, ATG | None | None |
| **Table footnote**:  † Death after 12.6 years following allo-HSCT due to his pancreatic neuroendocrine tumour.  **Abbreviations**:  SSM, secondary solid malignancy; TBI, total body irradiation; IS, systemic immunosuppression; acute GVHD, acute chronic graft-versus-host disease maximum staging; ITP, immune thrombocytopenia; PT-Cy/Tacro/MMF, post-transplant cyclophosphamide in combination with tacrolimus and mycophenolate mofetil; Cy, cyclophosphamide; Flu, fludarabine; CyA/MTX, cyclosporine and methotrexate; CyA/MMF, cyclosporine and mycophenolate mofetil; Tac/MMF, tacrolimus and mycophenolate mofetil; ATG, anti-thymocyte globulin. | | | | | | | | |
